# A New Ferroptosis-Related lncRNA Signature Predicts the Prognosis of Bladder Cancer Patients

**DOI:** 10.3389/fcell.2021.699804

**Published:** 2021-11-16

**Authors:** Mei Chen, Zhenyu Nie, Yan Li, Yuanhui Gao, Xiaohong Wen, Hui Cao, Shufang Zhang

**Affiliations:** Central Laboratory, Affiliated Haikou Hospital of Xiangya Medical College, Central South University, Haikou, China

**Keywords:** ferroptosis, lncRNAs, bladder cancer, immune infiltration, drug therapy

## Abstract

**Background:** Ferroptosis is closely related to the occurrence and development of cancer. An increasing number of studies have induced ferroptosis as a treatment strategy for cancer. However, the predictive value of ferroptosis-related lncRNAs in bladder cancer (BC) still need to be further elucidated. The purpose of this study was to construct a predictive signature based on ferroptosis-related long noncoding RNAs (lncRNAs) to predict the prognosis of BC patients.

**Methods:** We downloaded RNA-seq data and the corresponding clinical and prognostic data from The Cancer Genome Atlas (TCGA) database and performed univariate and multivariate Cox regression analyses to obtain ferroptosis-related lncRNAs to construct a predictive signature. The Kaplan-Meier method was used to analyze the overall survival (OS) rate of the high-risk and low-risk groups. Gene set enrichment analysis (GSEA) was performed to explore the functional differences between the high- and low-risk groups. Single-sample gene set enrichment analysis (ssGSEA) was used to explore the relationship between the predictive signature and immune status. Finally, the correlation between the predictive signature and the treatment response of BC patients was analyzed.

**Results:** We constructed a signature composed of nine ferroptosis-related lncRNAs (AL031775.1, AL162586.1, AC034236.2, LINC01004, OCIAD1-AS1, AL136084.3, AP003352.1, Z84484.1, AC022150.2). Compared with the low-risk group, the high-risk group had a worse prognosis. The ferroptosis-related lncRNA signature could independently predict the prognosis of patients with BC. Compared with clinicopathological variables, the ferroptosis-related lncRNA signature has a higher diagnostic efficiency, and the area under the receiver operating characteristic curve was 0.707. When patients were stratified according to different clinicopathological variables, the OS of patients in the high-risk group was shorter than that of those in the low-risk group. GSEA showed that tumor- and immune-related pathways were mainly enriched in the high-risk group. ssGSEA showed that the predictive signature was significantly related to the immune status of BC patients. High-risk patients were more sensitive to anti-PD-1/L1 immunotherapy and the conventional chemotherapy drugs sunitinib, paclitaxel, cisplatin, and docetaxel.

**Conclusion:** The predictive signature can independently predict the prognosis of BC patients, provides a basis for the mechanism of ferroptosis-related lncRNAs in BC and provides clinical treatment guidance for patients with BC.

## Introduction

Bladder cancer (BC) is the ninth most common malignant cancer in the world and the most common tumor in the urinary system (Bray et al., 2018). BC is divided into muscle-invasive BC (MIBC) and non-MIBC (NMIBC); 75% of patients are NMIBC, and 25% are MIBC (Witjes et al., 2021). The 5-years survival rate of NMIBC is more than 90% (Knowles and Hurst, 2015). Approximately 50–70% of patients with NMIBC will relapse, and approximately 10–20% will develop MIBC (Rübben et al., 1988). The 5-years survival rate of patients with MIBC is less than 50% (Alfred-Witjes et al., 2017). Progress has been made in the treatment of BC with the emergence of immune checkpoint inhibitors. For advanced BC, cisplatin-based combination chemotherapy is the first-line treatment option. The high recurrence rate and drug resistance phenotype of BC are present difficulties in clinical research. Therefore, looking for predictive markers of BC could be of great significance for monitoring the recurrence of BC and revealing new therapeutic targets.

Ferroptosis is an iron-dependent form of nonapoptotic cell death (Dixon et al., 2012). Ferroptosis is a newly discovered form of cell death caused by the accumulation of intracellular iron and lipid reactive oxygen species (Hirschhorn and Stockwell, 2019). Ferroptosis is mainly marked by a significant increase in cytoplasmic iron and lipid reactive oxygen species, a decrease in the volume of mitochondria, and an increase in the thickness of the bilayer membrane (Xie et al., 2016). Recent studies have shown that ferroptosis is closely related to the occurrence and development of cancer, and the induction of ferroptosis has become a new type of tumor treatment. miR-324-3p reverses cisplatin resistance by inducing GPX4-mediated ferroptosis in lung adenocarcinoma (Deng et al., 2021). SUV39H1 deficiency inhibits the growth of clear cell renal cell carcinoma by inducing ferroptosis (Wang et al., 2021). Silencing PTPN18 can induce ferroptosis in endometrial cancer through p-P38-mediated downregulation of GPX4/xCT (Wang et al., 2021). Apatinib promotes ferroptosis in colorectal cancer by targeting ELOVL6/ACSL4 (Tian et al., 2021). Inhibition of GPX4 can induce ferroptosis and enhance the sensitivity of triple-negative breast cancer to gefitinib (Song et al., 2020).

Long noncoding RNAs (lncRNAs) are noncoding RNAs with lengths of more than 200 nucleotides. LncRNAs regulates tumorigenesis (Schmitt and Chang, 2016). ADAMTS9-AS2 regulates the proliferation, migration and apoptosis of BC cells by targeting miR-182-5p (Guo et al., 2021). The LINC01140/miR-140-5p/FGF9 axis regulates the invasiveness of BC cells and the M2 polarization of macrophages (Wu et al., 2020). LncRNA PVT1 accelerates the malignant phenotype of BC cells by regulating the miR-194-5p/BCLAF1 axis as a competing endogenous RNA (Chen et al., 2020). LncRNAs are critical factors in the chemoresistance of BC (Zangouei et al., 2020). At present, there are few studies on lncRNAs related to ferroptosis and the research on ferroptosis-related lncRNAs in BC has not been reported.

In this study, we constructed a predictive signature based on ferroptosis-related lncRNAs; analyzed its value for evaluating the prognosis, diagnosis, chemotherapy response and tumor immune infiltration of BC patients; and conducted internal verification. We further carried out gene enrichment analysis (GSEA) to the explore potential mechanisms.

## Materials and Methods

### Patients and Datasets

We downloaded the fragments per kilobase of transcript per million mapped reads (FPKM)-standardized RNA-seq data and the corresponding clinical and prognostic data for The Cancer Genome Atlas bladder cancer (TCGA-BLCA) dataset from the TCGA website (https://portal.gdc.cancer.gov/); data for 406 patients with lncRNA expression values and survival times were obtained. We obtained disease-free survival (DFS) data for 319 BC patients from the cBioPortal database (https://www.cbioportal.org/). A total of 259 ferroptosis-related genes (drivers: 108; suppressors: 69; markers: 111) were downloaded from FerrDb (Zhou and Bao, 2020).

### Functional Enrichment Analysis of Differentially Expressed Ferroptosis-Related Genes

We used a false discovery rate (FDR) < 0.05 and |log _2_ fold change (FC) > 1| as screening criteria to obtain ferroptosis-related differentially expressed genes (DEGs). We performed Gene Ontology (GO) and Kyoto Encyclopedia of Genes and Genomes (KEGG) analyses in the “ggplot2” package.

### Construction of the Ferroptosis-Related lncRNA Predictive Signature

We used the “limma” package to calculate the correlation between ferroptosis-related genes and lncRNAs. Using the correlation coefficient |*R* ^2^ | > 0.4 and *p* < 0.001 as the screening criteria, a total of 805 ferroptosis-related lncRNAs with expression values were obtained. We first used univariate Cox regression analysis to obtain ferroptosis-related lncRNAs related to the prognosis of BC patients, and then we carried out multivariate Cox regression analysis to obtain ferroptosis-related lncRNAs for constructing the ferroptosis-related lncRNA predictive signature. The computational formula used for this analysis was as follows:

$$\mathrm{Risk}\;\mathrm{score}=\sum_{\mathrm{i=1}}^{n}\left(C\mathrm{oe}f_{i}\times x_{i}\right)$$

Coef represents the coefficient value, and x represents the expression value of selected ferroptosis-related lncRNAs. This formula was used to calculate the risk score of each BC patient.

### Construction of Nomogram

We combined the risk score with the clinicopathological characteristics of age, sex, stage, N stage to construct a nomogram that can predict the 1-, 3-, and 5-years survival of BC patients. We used a calibration curve to test whether the predicted survival rate was consistent with the actual survival rate.

### Functional Enrichment Analysis of the Ferroptosis-Related lncRNA Predictive Signature

According to the median value of the risk score, BC patients were divided into high- and low-risk groups. GSEA was used to analyze which pathway genes were mainly enriched (Subramanian et al., 2005). GSEA was carried out with GSEA 4.1.0 (http://www.broad.mit.edu/gsea/). Nominal *p* < 0.05 and FDR <0.25 were considered the thresholds for statistical significance. The infiltration scores of 16 immune cells and the activities of 13 immune-related pathways were calculated using the “GSVA” package by single-sample gene set enrichment analysis (ssGSEA) (Rooney et al., 2015).

### The Role of the Predictive Signature in Predicting the Clinical Treatment Response

To evaluate the role of the predictive signature in predicting the response to BC treatment, we calculated the half-maximal inhibitory concentration (IC50) of common chemotherapy drugs applied for the clinical treatment of BC. The Wilcoxon signed-rank test was used to compare the IC50 values between the high- and low-risk groups.

### Statistical Analysis

All statistical analyses were performed with R software (Version 4.0.2). The Wilcoxon test was used to analyze the expression levels of ferroptosis-related DEGs in cancer tissues and normal tissues. Univariate Cox regression analysis was used to analyze the relationship between ferroptosis-related lncRNAs and overall survival (OS), and multivariate Cox analysis was used to screen ferroptosis-related lncRNAs to construct a predictive signature. The Kaplan-Meier method and log-rank test were used to analyze the OS of patients in the high- and low-risk groups. The “survivalROC” package was used to draw the receiver operating characteristic (ROC) curves and determine the area under the curve (AUC) values. The “GSVA” package was used for ssGSEA.

## Results

### Enrichment Analysis of Ferroptosis-Related Genes

**FIGURE 1:**
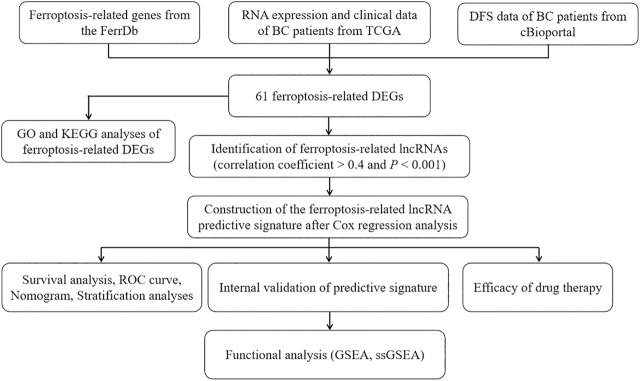
The flowchart of our research. BC, bladder cancer; TCGA, The Cancer Genome Atlas; DFS, disease-free survival; DEGs, differentially expressed genes; GO, Gene Ontology; KEGG, Kyoto Encyclopedia of Genes and Genomes; lncRNAs, long noncoding RNAs; ROC, receiver operating characteristic; GSEA, gene enrichment analysis; ssGSEA, single-sample gene set enrichment analysis.

**FIGURE 2:**
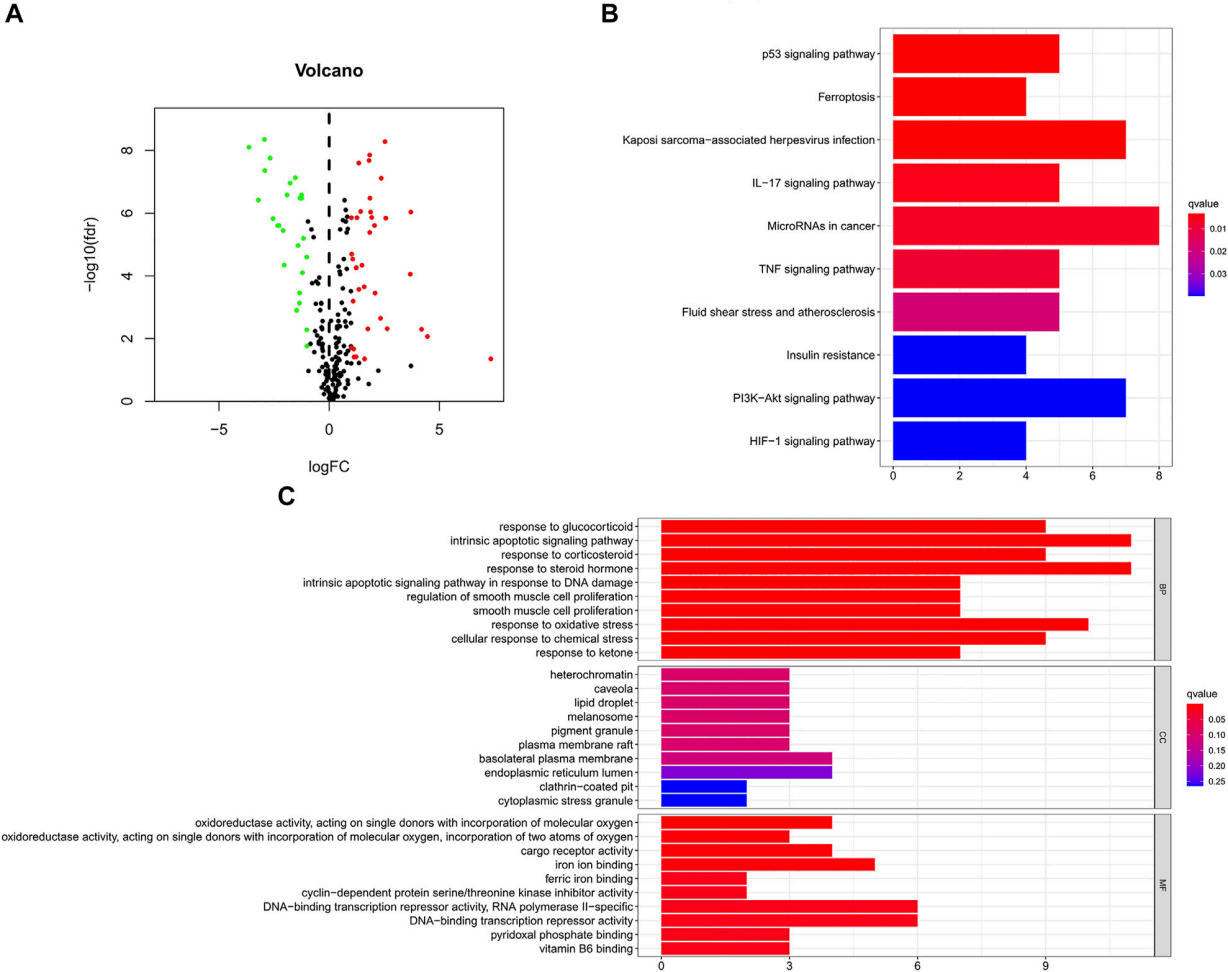
GO and KEGG analyses of ferroptosis-related DEGs in cancer and adjacent tissues. **(A)** Volcano plot of 259 ferroptosis-related genes in BC. Red dots represent up-regulated genes and green dots represent down-regulated genes. **(B)** KEGG analysis of ferroptosis-related DEGs. **(C)** GO analysis of ferroptosis-related DEGs. GO, Gene Ontology; KEGG, Kyoto Encyclopedia of Genes and Genomes; DEGs, differentially expressed genes; FC, fold change; fdr, false discovery rate; BP, biological process; CC, cellular components; MF, molecular function.

Figure 1 is a flowchart of our research. We obtained 61 ferroptosis-related DEGs, including 36 upregulated genes and 25 downregulated genes (Figure 2A). We performed KEGG and GO analyses of ferroptosis-related DEGs. KEGG pathway analyses indicated that ferroptosis-related DEGs were mainly enriched in the p53 signaling pathway, ferroptosis, kaposi sarcoma-associated herpesvirus infection, the IL-17 signaling pathway, microRNAs in cancer, the TNF signaling pathway, fluid shear stress and atherosclerosis, insulin resistance, the PI3K-Akt signaling pathway and the HIF-1 signaling pathway (Figure 2B). In the biological process category, GO analysis showed that DEGs were mainly enriched in the response to glucocorticoids, the intrinsic apoptotic signaling pathway, the response to oxidative stress, etc. In the cellular components category, the DEGs were mainly enriched in heterochromatin, melanosomes, the endoplasmic reticulum lumen, etc., In the molecular function category, the DEGs were mainly enriched in iron ion binding, ferric iron binding, DNA-binding transcription repressor activity, etc (Figure 2C).

### Construction of the Ferroptosis-Related lncRNA Predictive Signature

**FIGURE 3:**
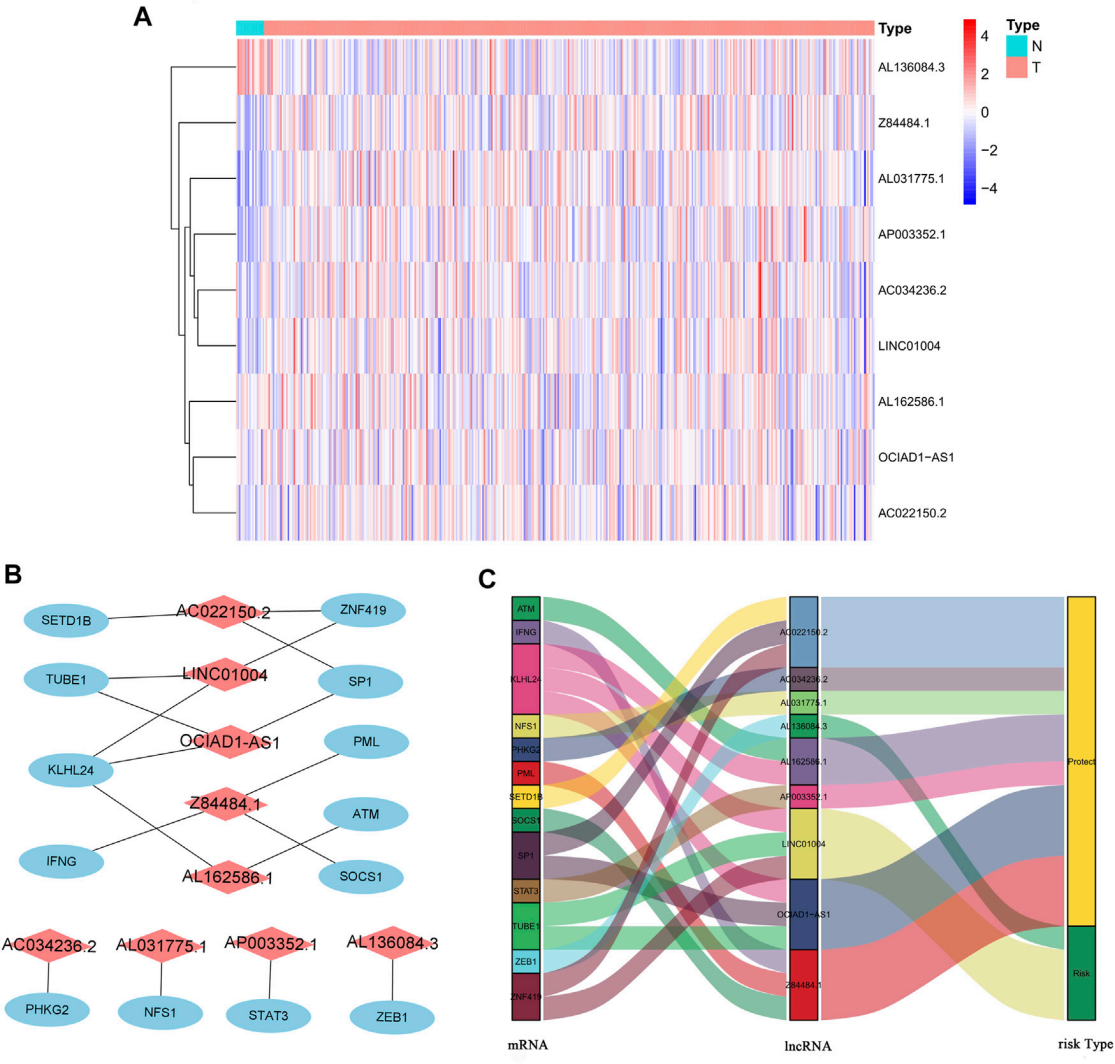
The expression levels and lncRNA-mRNA network of nine ferroptosis-related lncRNAs in the predictive signature. **(A)** The expression levels of nine ferroptosis-related lncRNAs in BC and normal tissues. **(B)** The co-expression network of prognostic ferroptosis-related lncRNAs. **(C)** Sankey diagram of prognostic ferroptosis-based lncRNAs. lncRNAs, long noncoding RNAs; BC, bladder cancer; N, normal; T, tumor.

We identified 805 ferroptosis-related lncRNAs (Supplementary Table S1). Univariate Cox regression analysis revealed that 33 lncRNAs were associated with the prognosis of BC patients. Multivariate Cox regression analysis revealed that 9 ferroptosis-related lncRNAs (AL031775.1, AL162586.1, AC034236.2, LINC01004, OCIAD1-AS1, AL136084.3, AP003352.1, Z84484.1, AC022150.2) were identified to construct a predictive signature. The expression levels of 9 ferroptosis-related lncRNAs in BC patients were shown in Figure 3A. We further visualized the lncRNAs with Cytoscape and the ggalluvial R software package. The co-expression network contained 18 pairs lncRNA-mRNA (Figure 3B, |*R* ^2^ | > 0.4 and *p* < 0.001). AC022150.2 had coexpressive relationship with three ferroptosis-related genes (SETD1B, ZNF419 and SP1), LINC01004 had coexpressive relationship with three ferroptosis-related genes (ZNF419, TUBE1 and KLHL24), OCIAD1-AS1 had coexpressive relationship with three ferroptosis-related genes (SP1, TUBE1 and KLHL24), Z84484.1 had coexpressive relationship with three ferroptosis-related genes (PML, IFNG and SOCS1), AL162586.1 had coexpressive relationship with two ferroptosis-related genes (KLHL24 and ATM), AC034236.2 was coexpressed with PHKG2, AL031775.1 was coexpressed with NFS1, AP003352.1 was coexpressed with STAT3, and AL136084.3 was coexpressed with ZEB1. AL031775.1, AL162586.1, AC034236.2, OCIAD1-AS1, AP003352.1, Z84484.1 and AC022150.2 were protective factors, while LINC01004 and AL136084.3 were risk factors (Figure 3C). The risk score was calculated as follows: risk score=(-0.251 × AL031775.1 expression)+(-0.204 × AL162586.1 expression)+(-0.250 × AC034236.2 expression)+(0.234×LINC01004 expression)+(-0.394×OCIAD1-AS1 expression)+(0.138 × AL136084.3 expression)+(-0.116 × AP003352.1 expression)+(-0.703 × Z84484.1 expression)+(-0.101 × AC022150.2 expression).

### Correlation Between the Predictive Signature and the Prognosis of BC Patients

**FIGURE 4:**
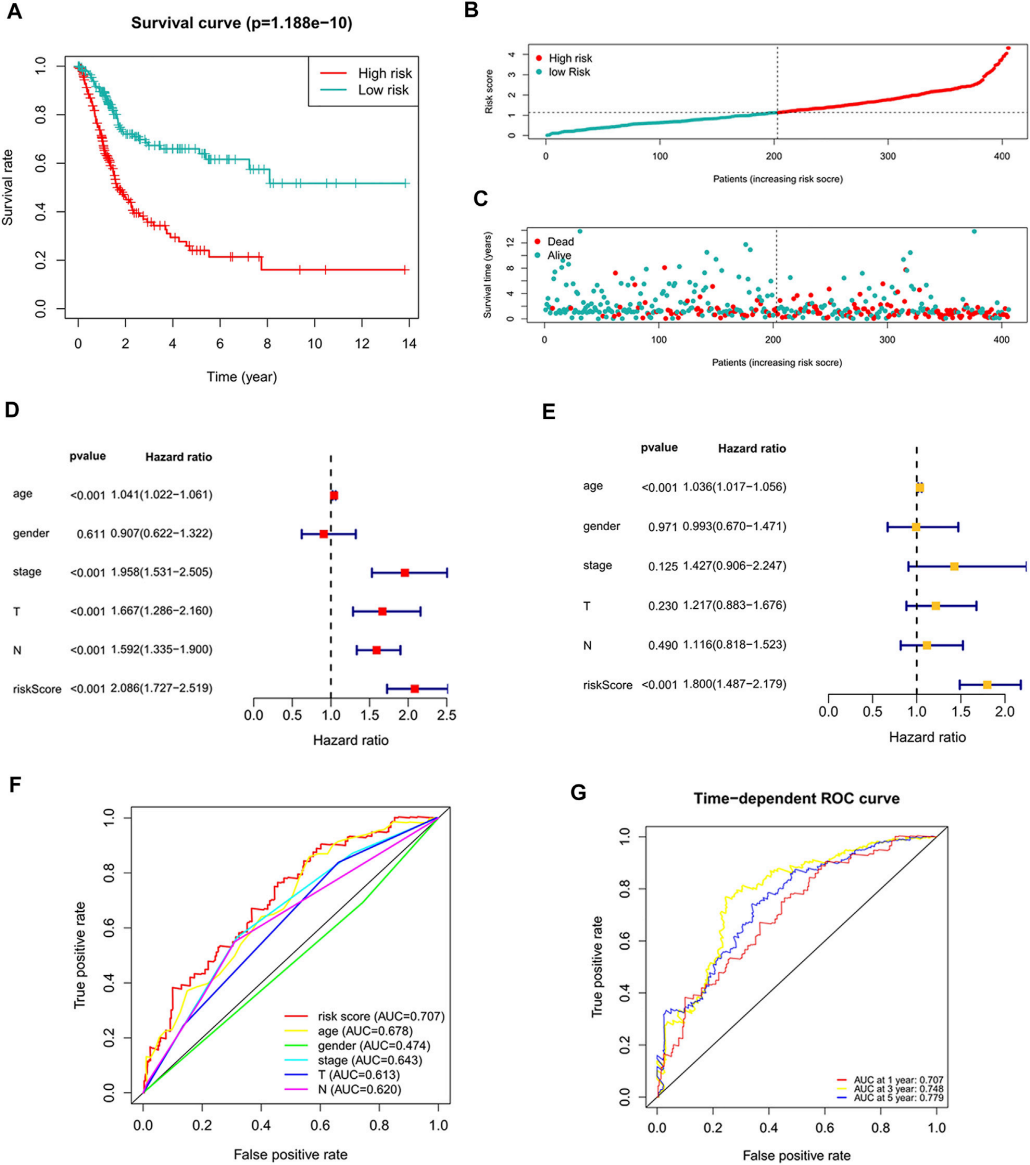
The correlation between the predictive signature and the prognosis of BC patients. **(A)** Kaplan-Meier analysis of the OS rate of BC patients in the high- and low-risk groups. **(B)** The distribution of the risk score among BC patients. **(C)** The number of dead and alive patients with different risk scores. Blue represents the number of survivors, and red represents the number of deaths. **(D)** Forest plot for univariate Cox regression analysis. **(E)** Forest plot for multivariate Cox regression analysis. **(F)** The ROC curve of the risk score and clinicopathological variables. **(G)** ROC curve and AUCs at 1-year, 3-years and 5-years survival for the predictive signature. BC, bladder cancer; OS, overall survival; ROC, receiver operating characteristic; AUC, area under the curve; T, tumor; N, lymph node.

**FIGURE 5:**
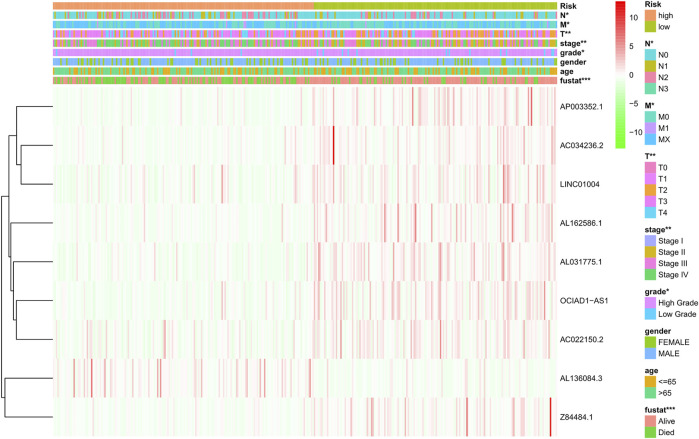
Distribution heat map of nine prognostic ferroptosis-related lncRNAs and clinicopathological variables in the high- and low-risk groups. lncRNAs, long noncoding RNAs; N, lymph node; M, metastasis; T, tumor.

The risk score of each patient was calculated according to the formula, and the patients were divided into high-risk and low-risk groups according to the median value of the risk score. To determine the value of the risk score in predicting the prognosis of BC patients, Kaplan-Meier analysis was used to analyze the OS time of the high- and low-risk groups. Compared with that of the low-risk group, the OS time of the high-risk group was significantly shorter (Figure 4A, *p* = 1.188e-10). The 5-years survival rates of the high- and low-risk groups were 24.1 and 63.9%, respectively. The risk scores of the high- and low-risk groups are shown in Figure 4B. With the increase of risk score, more and more patients died (Figure 4C). To determine whether the predictive signature is an independent prognostic factor for BC patients, Cox regression analysis was performed. Univariate Cox regression analysis showed that age, stage, T stage, N stage and risk score were significantly associated with the OS of BC patients (Figure 4D). Multivariate Cox regression analysis showed that age and risk score were independent predictors of OS in BC patients (Figure 4E). The AUC of the risk score was 0.707, which was better than that of clinicopathological variables in predicting the prognosis of BC patients (Figure 4F). The AUCs of 1, 3, and 5-years survival were 0.707, 0.748 and 0.779, respectively, which indicated good predictive performance (Figure 4G). We analyzed the differences in clinicopathological variables between the high- and low-risk groups and found that N stage (*p* < 0.05), M stage (*p* < 0.05), T stage (*p* < 0.01), stage (*p* < 0.01), grade (*p* < 0.05), and fustat (*p* < 0.001) were different between the high- and low-risk groups (Figure 5).

**FIGURE 6:**
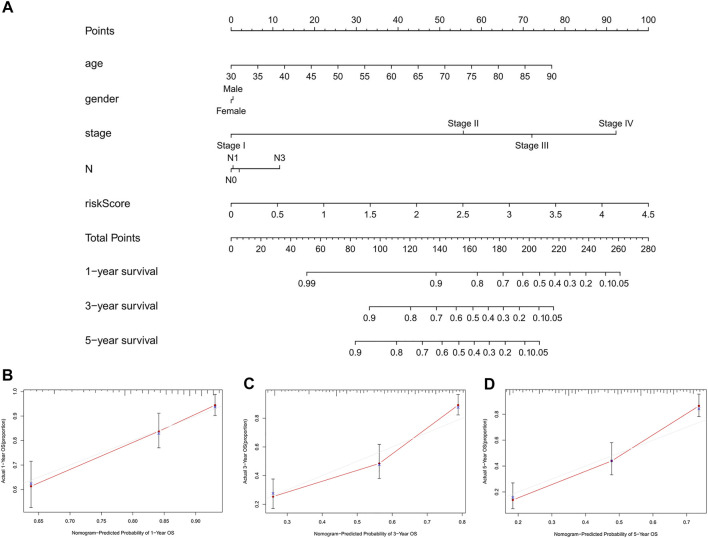
Construction and verification of the nomogram. **(A)** A nomogram combining clinicopathological variables and risk score predicts 1, 3, and 5 years OS of BC patients. **(B-D)** The calibration curves test consistency between the actual OS rates and the predicted survival rates at 1, 3 and 5 years. N, lymph node; OS, overall survival; BC, bladder cancer.

To further predict the prognosis of BC patients, we constructed a nomogram including clinicopathological variables and the risk score, and this nomogram could predict the 1, 3, and 5-years prognosis of BC patients (Figure 6A). The calibration curves showed good consistency between the actual OS rates and the predicted survival rates at 1, 3 and 5 years (Figures 6B–D).

### Relationship Between the Predictive Signature and the Prognosis of BC Patients in Different Clinicopathological Variables

**FIGURE 7:**
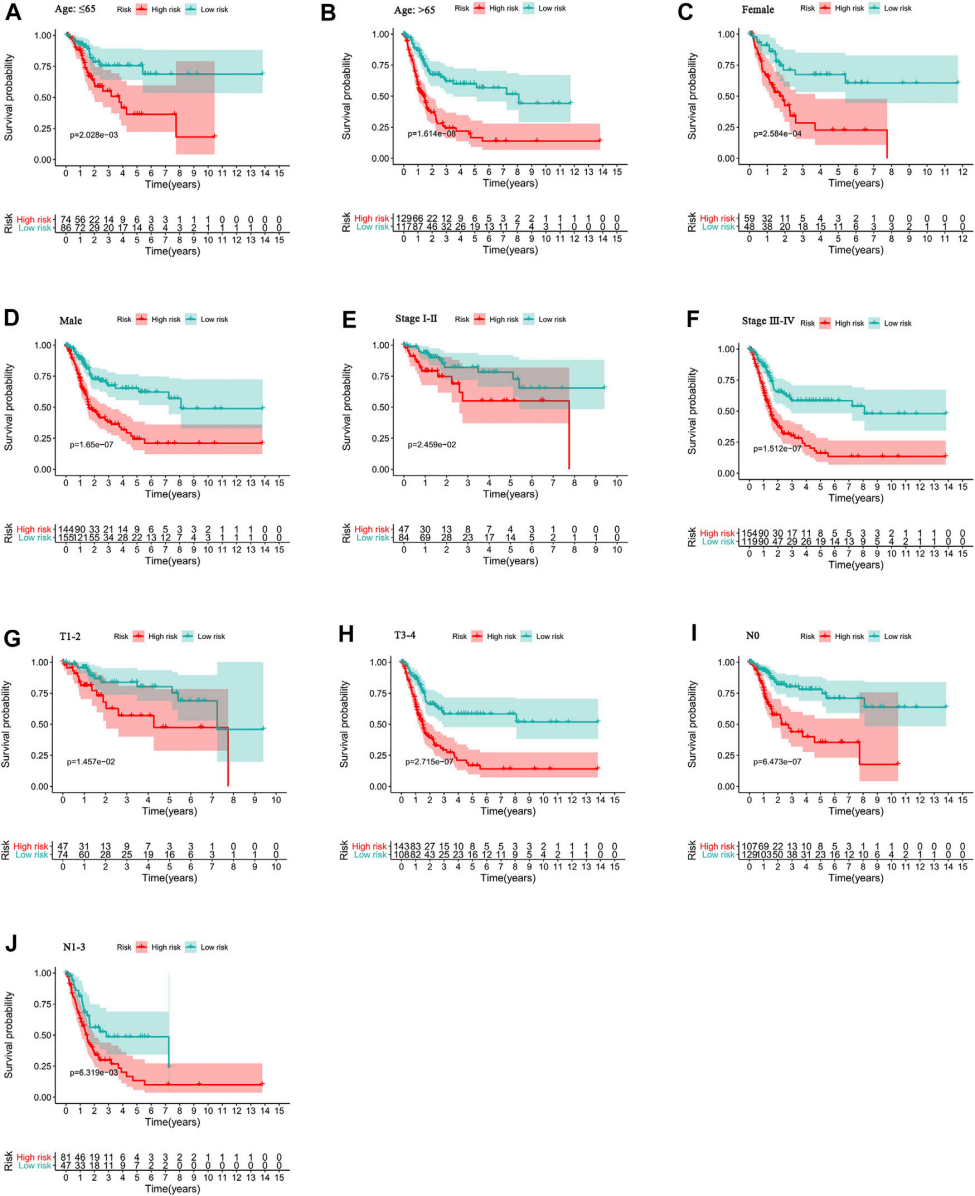
Kaplan-Meier survival curves of high- and low-risk groups among patients sorted according to different clinicopathological variables. **(A-B)** Age. **(C-D)** Sex. **(E-F)** Stage. **(G-H)** T stage. **(I-J)** N stage. T, tumor; N, lymph node.

To study the relationship between the predictive signature and the prognosis of BC patients sorted according to different clinicopathological variables, BC patients were separated into groups according to age, sex, stage, T stage and N stage. For each different classifications, the OS of patients in the high-risk group was significantly shorter than that of patients in the low-risk group (Figure 7). These results suggest that the predictive signature can predict the prognosis of BC patients without considering clinicopathological variables.

### Internal Validation of the Predictive Signature

**FIGURE 8:**
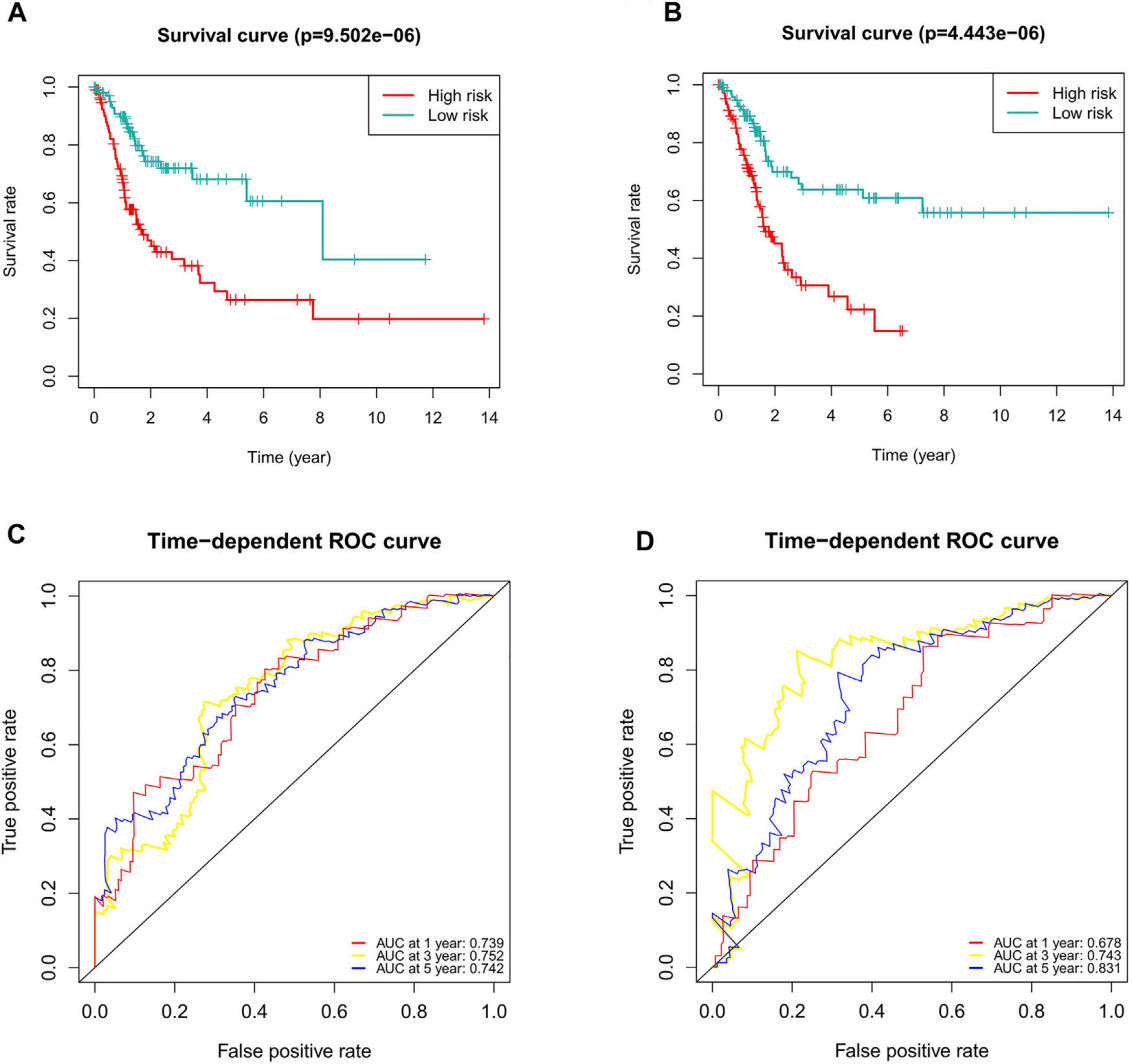
Internal validation of the predictive signature for OS based on the entire TCGA dataset. **(A)** Kaplan-Meier survival curve in the first internal cohort. **(B)** Kaplan-Meier survival curve in the second internal cohort. **(C)** ROC curve and AUCs at 1-year, 3-years and 5-years survival in the first internal cohort. **(D)** ROC curve and AUCs at 1-year, 3-years and 5-years survival in the second internal cohort. ROC, receiver operating characteristic; AUC, area under the curve; OS, overall survival; TCGA, The Cancer Genome Atlas.

To verify the applicability of the predictive signature for OS based on the entire TCGA dataset, we randomly divided the 406 BC patients into two cohorts (n = 203). The demographic characteristics of patients in the two cohorts are shown in Table 1. Consistent with the results observed in the entire dataset, the OS rate of patients in the high-risk group was lower than that of the low-risk group in the first internal cohort (Figure 8A, *p* = 9.502e-06). In the second internal cohort, the prognosis of the high-risk group was worse than that of the low-risk group (Figure 8B, *p* = 4.443e-06). The ROC curves of two cohorts showed good predictive performance. In the first internal cohort, the AUCs of 1, 3, and 5-years survival were 0.739, 0.752, and 0.742, respectively (Figure 8C). In the second internal cohort, the AUCs of 1, 3, and 5-years survival were 0.678, 0.743, and 0.831, respectively (Figure 8D).

**TABLE 1:** The clinical characteristics of patients in different cohorts.

Variables	Entire TCGA dataset (n = 406)	Validation cohort	
		First cohort (n = 203) second cohort (n = 203)	
Age (%)			
≤65	161 (39.7)	78 (38.4)	83 (40.9)
>65	245 (60.3)	125 (61.6)	120 (59.1)
Gender (%)			
Female	108 (26.5)	54 (26.6)	54 (26.6)
Male	298 (73.4)	149 (73.4)	149 (73.4)
Grade (%)			
Low	20 (4.9)	8 (3.9)	12 (5.9)
High	383 (94.3)	193 (95.1)	190 (93.6)
Unknow	3 (0.8)	2 (1.0)	1 (0.5)
Stage (%)			
I + II	131 (32.3)	58 (28.6)	73 (36.0)
III + IV	273 (67.2)	142 (70.4)	130 (64.0)
Unknow	2 (0.5)	2 (1.0)	0 (0.0)
T (%)			
T0	1 (0.2)	1 (0.5)	0 (0.0)
T1+T2	130 (29.8)	56 (27.6)	65 (32.0)
T3+T4	251 (61.9)	134 (66.0)	117 (57.7)
TX + Unknow	33 (8.1)	12 (5.9)	21 (10.3)
M (%)			
M0	195 (48.1)	100 (49.3)	95 (46.8)
M1	11 (2.7)	4 (2.0)	7 (3.4)
MX + Unknow	200 (49.2)	99 (48.7)	101 (49.8)
N (%)			
N0	236 (58.1)	114 (56.2)	122 (60.1)
N1+N2	121 (29.8)	69 (33.9)	52 (25.6)
N3	7 (1.7)	3 (1.5)	4 (2.0)
NX + Unknow	42 (10.4)	17 (8.4)	25 (12.3)

T, tumor; M, metastasis; N, lymph node.

### Gene Enrichment Analysis

Because of the different prognoses of patients in the high- and low-risk groups, we conducted GSEA to study the possible differences between the high- and low-risk groups. We found that BC, MAPK signaling pathway, chemokine signaling pathway, T/B cell receptor signaling pathway and Fc gamma R mediated phagocytosis were significantly enriched in the high-risk group (Supplementary Figures S1A–F, Table 2), indicating that high-risk patients are closely related to tumor- and immune-related pathways.

**TABLE 2:** The high-risk group enriched gene sets.

Gene set	ES	NES	NOM *p*-val	FDR *q*-val
Bladder cancer	0.56	2.01	0.004	0.005
MAPK signaling pathway	0.50	2.13	0.000	0.002
Chemokine signaling pathway	0.57	2.15	0.000	0.002
T cell receptor signaling pathway	0.50	1.80	0.022	0.026
B cell receptor signaling pathway	0.53	1.77	0.020	0.032
Fc gamma R mediated phagocytosis	0.47	1.86	0.006	0.017

ES, enrichment score; NES, normalized enrichment score; FDR, false discovery rate; MAPK, mitogen-activated protein kinase.

### Immune Cell Infiltration and Immune-Related Pathways

**FIGURE 9:**
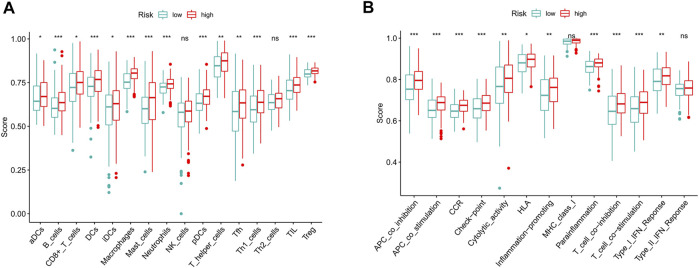
The scores of immune infiltrating cells and immune-related functions in high- and low-risk groups. **(A)** ssGSEA algorithm was used to calculate the infiltration levels of 16 immune cells in high- and low-risk groups. **(B)** The correlation between the predictive signature and 13 immune-related functions. ssGSEA, single-sample gene set enrichment analysis; aDCs, activated dendritic cells; iDCs, immature dendritic cells; NK, natural killer; pDCs, plasmacytoid dendritic cells; Tfh, T follicular helper; Th1, T helper type 1; Th2, T helper type 2; TIL, tumor-infiltrating lymphocyte; Treg, T regulatory cell; APC, antigen-presenting cell; CCR, chemokine receptor; HLA, human leukocyte antigen; MHC, major histocompatibility complex; IFN, interferon. *p < 0.05; **p < 0.01; ***p < 0.001; ns, non-significant.

To further explore the correlation between risk scores and immune cells and functions, we quantified the enrichment scores of ssGSEA for different immune cell subgroups, related functions or pathways. The results showed that activated dendritic cells (aDCs), B cells, CD8^+^ T cells, DCs, immature dendritic cells (iDCs), macrophages, mast cells, neutrophils, plasmacytoid dendritic cells (pDCs), T helper cells, T follicular helper (Tfh) cells, T helper type 1 (Th1) cells, tumor-infiltrating lymphocyte (TIL) and T regulatory cells (Tregs) were significantly different in the high- and low-risk groups (Figure 9A). The immune function scores of antigen-presenting cell (APC) coinhibition, APC costimulation, chemokine receptor (CCR), checkpoint, cytolytic activity, human leukocyte antigen (HLA), inflammation promoting, parainflammation, T cell coinhibition, T cell costimulation and type Ⅰ IFN response were higher in the high-risk group than in the low-risk group (Figure 9B). These findings suggest that immunological function is more active in the high-risk group.

### Correlation Between the Predictive Signature and BC Therapy

**FIGURE 10:**
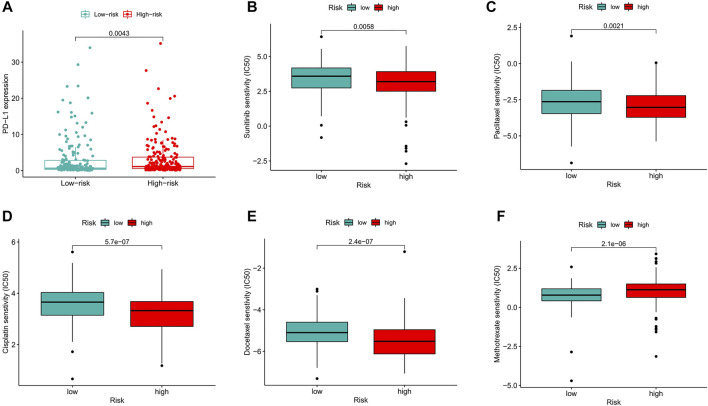
Comparison of treatment drugs senstivity between high- and low-risk groups. **(A)** PD-L1 expression in high and low risk groups. **(B)** IC50 of sunitinib in high and low risk groups. **(C)** IC50 of paclitaxel in high and low risk groups. **(D)** IC50 of cisplatin in high and low risk groups. **(E)** IC50 of docetaxel in high and low risk groups. **(F)** IC50 of methotrexate in high and low risk groups. IC50, half-maximal inhibitory concentration. PD-L1, programmed cell death ligand 1.

Compared with that in the low-risk group, PD−L1 expression in the high-risk group was significantly higher, suggesting that high-risk patients have a potential response to anti-PD-1/L1 immunotherapy (Figure 10A). In addition to immunotherapy, we also analyzed the association between the predictive signature and the efficacy of general chemotherapy for BC. The results found that the IC50 of sunitinib, paclitaxel, cisplatin, and docetaxel was lower in the high-risk group, and the IC50 of methotrexate in the high-risk group was higher (Figures 10B–F), which is helpful for exploring individualized treatment schemes suitable for high- and low-risk group patients.

### Construction of the Ferroptosis-Related lncRNA Predictive Signature for DFS

**FIGURE 11:**
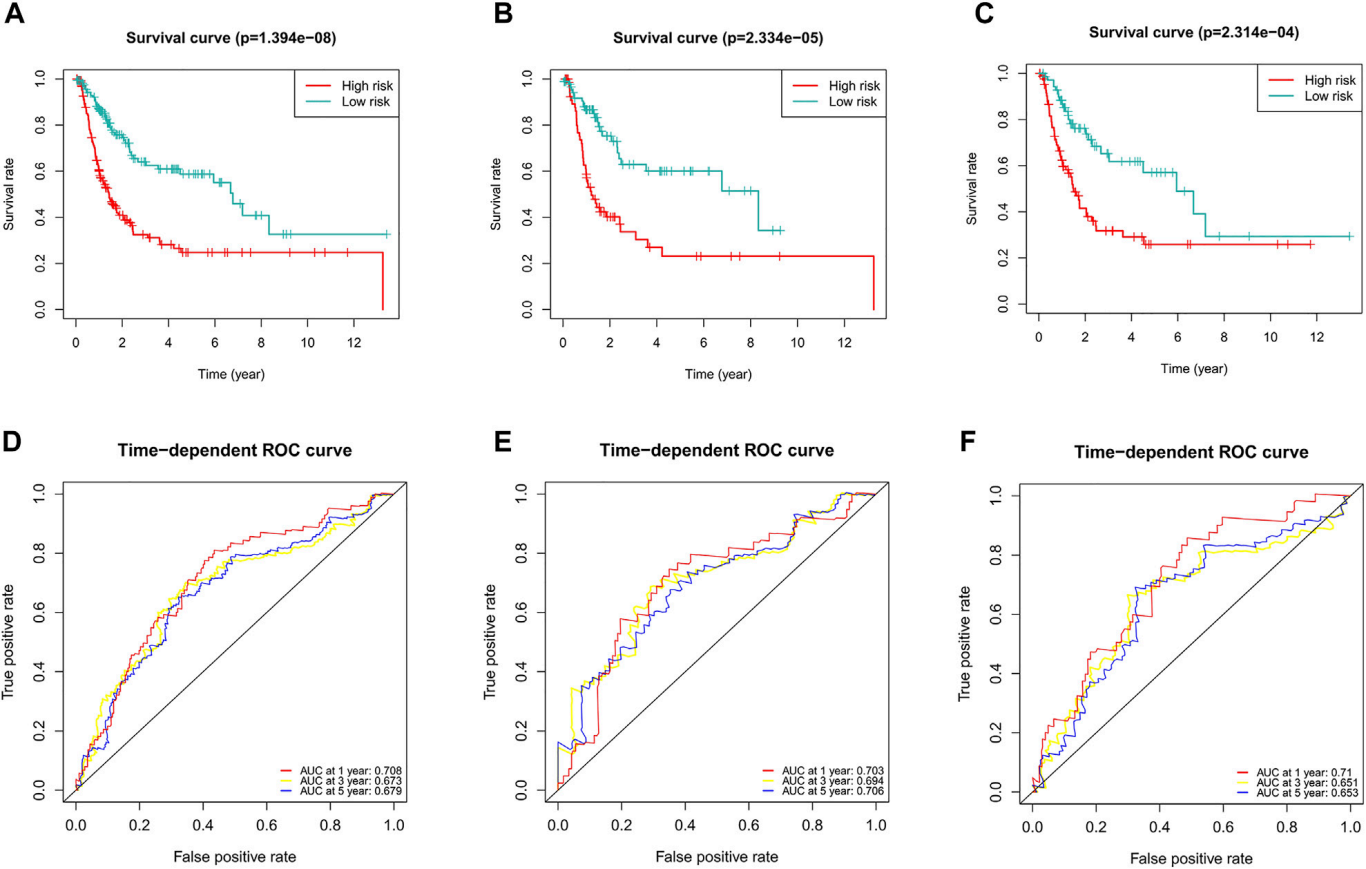
Evaluation of the predictive value of the ferroptosis-related lncRNA signature for DFS. **(A)** Kaplan-Meier survival curve in the entire dataset. **(B)** Kaplan-Meier survival curve in the first cohort. **(C)** Kaplan-Meier survival curve in the second cohort. **(D)** ROC curve and AUCs at 1-year, 3-years and 5-years survival in the entire dataset. **(E)** ROC curve and AUCs at 1-year, 3-years and 5-years survival in the first cohort. **(F)** ROC curve and AUCs at 1-year, 3-years and 5-years survival in the second cohort. lncRNAs, long noncoding RNAs; DFS, disease-free survival; ROC, receiver operating characteristic; AUC, area under the curve.

Considering the role of DFS in the prognosis of BC patients, we also constructed a ferroptosis-related lncRNA predictive signature for DFS. We collected the DFS data of BC patients from the cBioPortal database, including 319 patients. After univariate Cox regression analysis, we found that 16 ferroptosis-related lncRNAs were significantly associated with DFS in BC patients. After multivariate Cox regression analysis, three ferroptosis-related lncRNAs were obtained to construct the predictive signature. Risk score =(-0.096 × *AC104825.1*)+(-0.129 × *AL355472.1*)+(-0.146 × *AC011468.1*). The risk score of each patient was calculated according to the formula, and the patients in the entire dataset were divided into a high-risk group and a low-risk group according to the median value. Kaplan-Meier survival curve analysis showed that the DFS of the high-risk group was significantly shorter than that of the low-risk group (Figure 11A, *p* = 1.394e-08). The AUCs of 1, 3, and 5-years survival were 0.708, 0.673, and 0.679, respectively (Figure 11D).

To verify the applicability of the predictive signature for DFS, 319 patients were randomly divided into the first internal cohort (n = 160) and the second internal cohort (n = 159). Patients were divided into high- and low-risk groups based on the median value, and the results observed from the entire dataset were consistent. Compared with the low-risk group, the DFS of patients in the high-risk group was shorter in the first internal cohort (Figure 11B, *p* = 2.334e-05) and the second internal cohort (Figure 11C, *p* = 2.314e-04). In the first internal cohort, the AUCs of 1, 3, and 5-years survival were 0.703, 0.694, and 0.706, respectively (Figure 11E). In the second internal cohort, the AUCs of 1, 3, and 5-years survival were 0.71, 0.651, and 0.653, respectively (Figure 11F).

## Discussion

BC is one of the most common malignant tumors of the genitourinary system, most of which originate from the urothelium. The role of ferroptosis in cancer is complex. An increasing number of studies have found that ferroptosis plays a key role in the development and progression of cancer, but the current research focuses on the role of ferroptosis in cancer treatment (Chen et al., 2021; Ghoochani et al., 2021; Hou et al., 2021; Li et al., 2021; Luo et al., 2021). There are few studies on its role in cancer prognosis. Recently, studies have predicted the prognosis of cancer patients by constructing ferroptosis-related lncRNA predictive signatures (Gao et al., 2021; Jiang et al., 2021; Li et al., 2021; Liu et al., 2021; Luo et al., 2021; Yang et al., 2021; Zheng et al., 2021; Zhu et al., 2021). However, it has not been reported to predict the prognosis of BC patients by constructing ferroptosis-related lncRNA predictive signature.

In this study, we first obtained 61 ferroptosis-related DEGs. Then, KEGG analysis showed that the DEGs were mainly enriched in the p53 signaling pathway, ferroptosis, the IL-17 signaling pathway, microRNAs in cancer, the TNF signaling pathway, insulin resistance, the PI3K-Akt signaling pathway and the HIF-1 signaling pathway. Studies have shown that p53 inhibits the uptake of cystine and induces ferroptosis by inhibiting the expression of SLC7A11 (Jiang et al., 2015). Amy et al. (Tarangelo et al., 2018) found that p53 induces ferroptosis in cancer cells by inhibiting metabolic stress. These results indicate that ferroptosis-related genes may regulate the progression of BC through the p53 signaling pathway. However, further experiments are needed to verify the role of ferroptosis-related genes in BC.

Many studies have shown that lncRNAs play an important role in BC (Hu et al., 2021; Shi et al., 2021; Yang et al., 2021). In addition, many studies have also shown that lncRNAs play an important role in ferroptosis (Mao et al., 2018; Qi et al., 2019; Wang et al., 2019; Yang et al., 2020). LINC00618 accelerates ferroptosis by increasing the levels of lipid reactive oxygen and iron in leukemia and decreases the expression of SLC7A11, which accelerates ferroptosis by inducing apoptosis (Wang et al., 2021). Autophagy-related lncRNA signature can accurately predict the prognosis of BC patients (Sun et al., 2020). Ferroptosis-related lncRNAs can well predict the prognosis of colon cancer patients (Cai et al., 2021). Therefore, it is of great significance to identify a ferroptosis-related lncRNA predictive signature in BC patients. In this study, we used univariate Cox regression analysis to analyze the relationship between ferroptosis-related lncRNAs and the prognosis of BC patients and found that 33 lncRNAs were associated with the prognosis of BC patients. After multivariate Cox regression analysis, we identified nine ferroptosis-related lncRNAs (AL031775.1, AL162586.1, AC034236.2, LINC01004, OCIAD1-AS1, AL136084.3, AP003352.1, Z84484.1, AC022150.2) for inclusion in a predictive signature. We also found mRNA (SETD1B, TUBE1, KLHL24, IFNG, ZNF419, SP1, PML, ATM, SOCS1, PHKG2, NFS1, STAT3, and ZEB1) significantly co-expressed with these lncRNAs. Among them, PHKG2 is an important driving factor in the lipid peroxidation process of ferroptosis (Yang et al., 2016). Kinome screen of ferroptosis finds ATM plays an important role in iron metabolism (Chen et al., 2020). SOCS1 is involved in cell ferroptosis by regulating p53 expression (Saint-Germain et al., 2017). Inhibition of NFS1 can induce ferroptosis and slow tumor growth in lung adenocarcinoma (Alvarez et al., 2017). STAT3 is the target of ferroptosis in cancer cells, and impaired STAT3 signaling induces ferroptosis in diffuse large B-cell lymphoma cells (Chen et al., 2021; Schmitt et al., 2021). Each patient’s risk score was calculated according to the formula. The patients were divided into high-risk and low-risk groups according to the median value. The OS time of the high-risk group was shorter than that of the low-risk group. The ROC curve showed that the predictive signature had good predictive performance. The predictive signature was more reliable than clinicopathological variables in predicting the prognosis of BC patients. We also found that the predictive signature can predict the prognosis of BC patients without considering clinicopathological variables. Internal verification confirmed that the predictive signature has good predictive performance.

GSEA showed that BC, the MAPK signaling pathway, the chemokine signaling pathway, the T/B cell receptor signaling pathway, and FC gamma R-mediated phagocytosis were mainly enriched in the high-risk group. As a tumor promoter, RAB14 regulates the invasion and metastasis of BC cells by activating the MAPK signaling pathway (Chao et al., 2019). Upregulation of PKM2 may promote the proliferation, migration and invasion of BC cells through the MAPK signaling pathway (Hong et al., 2018). Maslinic acid induces apoptosis of BC cells through the p38 MAPK signaling pathway (Zhang et al., 2014). The GSEA results showed that high-risk patients are closely related to tumor- and immune-related pathways. The results of subsequent ssGSEA showed that CD8^+^ T cells, macrophages, mast cells, neutrophils, and Tregs had higher scores in the high-risk group. Studies have shown that high CD8^+^ T cell infiltration is associated with a poor prognosis in BC patients (Hou et al., 2020; Liu et al., 2020). High infiltration of tumor-associated macrophages is associated with a poor prognosis in advanced thyroid cancer (Ryder et al., 2008). The more resting mast cells there are, the worse the prognosis of prostate cancer patients is (Zhang et al., 2020). A high neutrophil-to-lymphocyte ratio predicts poor OS for patients with BC (Ohtake et al., 2016; Tan et al., 2018). High infiltration of Tregs is a poor prognostic indicator in patients with hepatocellular carcinoma (Tu et al., 2016). In addition to increased tumor immune cell infiltration, the high-risk group was associated with decreased antitumor immunity, with higher scores for HLA and the type Ⅰ IFN response. Therefore, the decreased antitumor immunity in the high-risk group may be the cause of the poor prognosis. Our research also shows that high-risk patients are probably sensitive to anti-PD-1/L1 immunotherapy and the conventional chemotherapy drugs sunitinib, paclitaxel, cisplatin, and docetaxel but are resistant to methotrexate. This indicates that the high-risk group of patients can benefit from the combination of immunotherapy and chemotherapy, which provides the basis for precise and personalized treatment for BC patients.

However, our research has some limitations. First, we only used the TCGA database data for internal validation, and we still need data from other databases for external validation to test the applicability of the predictive signature. Second, the mechanism of the ferroptosis-related lncRNAs in BC needs to be further verified by experiments.

In conclusion, the ferroptosis-related lncRNA signature can independently predict the prognosis of BC patients and provide the basis for the possible mechanism of ferroptosis-related lncRNAs in BC and the response to clinical treatment, but it still needs further experimental verification in the future.

## Data Availability

Publicly available datasets were analyzed in this study. The names of the repository/repositories and accession number(s) can be found in the article/Supplementary Material.
