# Supplementary figures and images for: A New Ferroptosis-Related lncRNA Signature Predicts the Prognosis of Bladder Cancer Patients
Source: Front Cell Dev Biol. 2021 Nov 16;9:699804. doi: 10.3389/fcell.2021.699804 (PMC8635160; doi:10.3389/fcell.2021.699804)

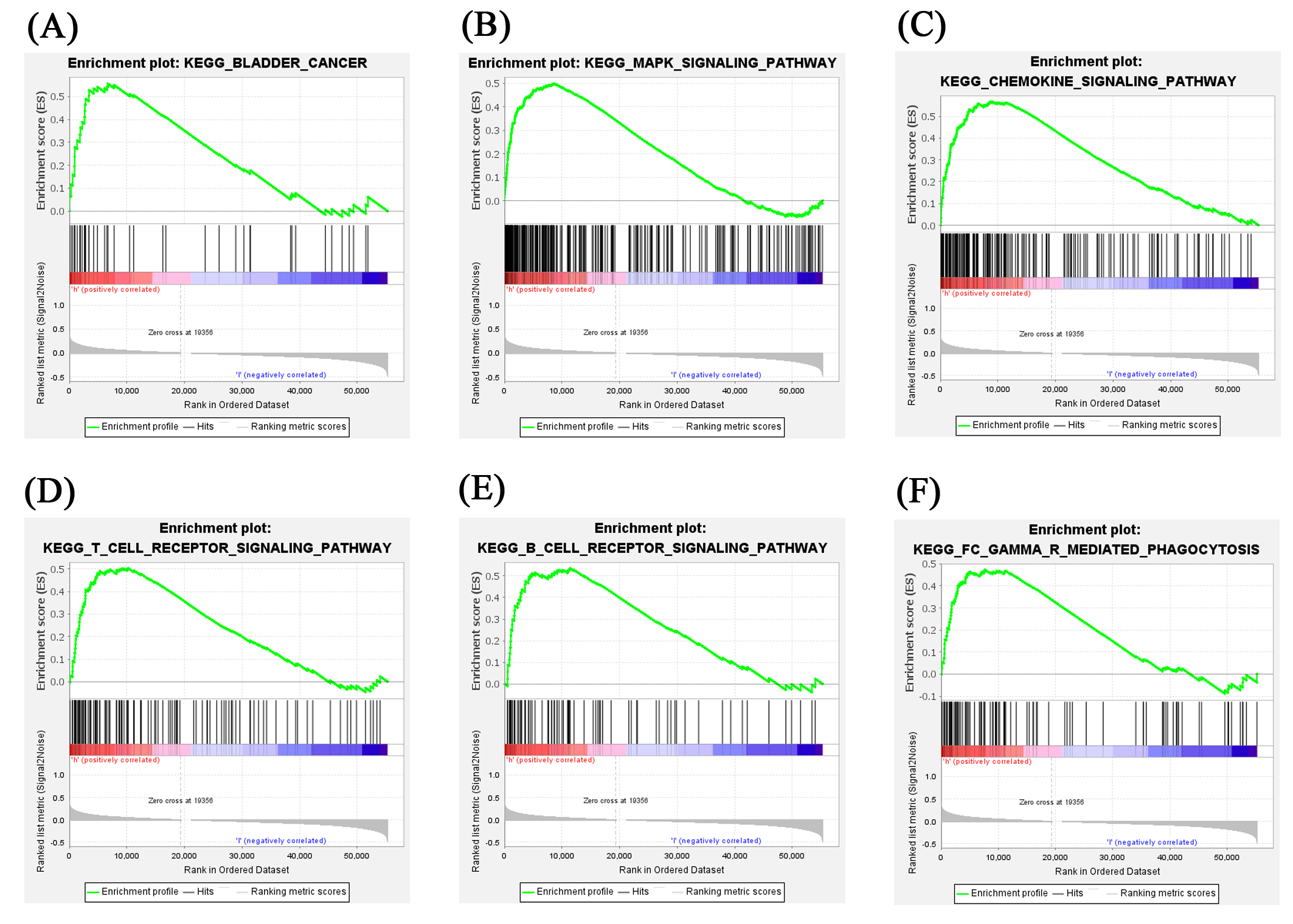

Supplement: Supplementary file 1 [file Image1.TIF]
